# Pooled Resequencing of 122 Ulcerative Colitis Genes in a Large Dutch Cohort Suggests Population-Specific Associations of Rare Variants in *MUC2*

**DOI:** 10.1371/journal.pone.0159609

**Published:** 2016-08-04

**Authors:** Marijn C. Visschedijk, Rudi Alberts, Soren Mucha, Patrick Deelen, Dirk J. de Jong, Marieke Pierik, Lieke M. Spekhorst, Floris Imhann, Andrea E. van der Meulen-de Jong, C. Janneke van der Woude, Adriaan A. van Bodegraven, Bas Oldenburg, Mark Löwenberg, Gerard Dijkstra, David Ellinghaus, Stefan Schreiber, Cisca Wijmenga, The Initiative on Crohn and Colitis, Parelsnoer Institute, Manuel A. Rivas, Andre Franke, Cleo C. van Diemen, Rinse K. Weersma

**Affiliations:** 1 Department of Gastroenterology and Hepatology, University of Groningen, University Medical Centre Groningen, 9700 RB, Groningen, The Netherlands; 2 Department of Genetics, University of Groningen, University Medical Centre Groningen, Groningen, 9700 RB, Groningen, The Netherlands; 3 Institute of Clinical Molecular Biology, Kiel University, D-24105, Kiel, Germany; 4 Department of Gastroenterology and Hepatology, Radboud University Nijmegen Medical Centre, 6525 GA, Nijmegen, The Netherlands; 5 Division of Gastroenterology and Hepatology, Maastricht University Medical Centre, 6229 HX, Maastricht, The Netherlands; 6 Department of Gastroenterology and Hepatology, Leiden University Medical Centre, 2333 ZA, Leiden, The Netherlands; 7 Department of Gastroenterology and Hepatology, Erasmus University Medical Centre, 3015 CE, Rotterdam, The Netherlands; 8 Department of Gastroenterology and Hepatology, VU University Medical Centre, 1081 HZ, Amsterdam, The Netherlands; 9 Department of Gastroenterology and Hepatology, University Medical Centre Utrecht, 3584 CX, Utrecht, The Netherlands; 10 Department of Gastroenterology and Hepatology, Academic Medical Centre, 1105 AZ, Amsterdam, The Netherlands; 11 Department of Internal Medicine I, University Medical Centre Schleswig-Holstein, Kiel, Germany; 12 Centre for the Study of IBD (SCIBD) Genetics, The Broad Institute, Cambridge, Massachusetts, United States of America; National Cancer Institute, National Institutes of Health, UNITED STATES

## Abstract

Genome-wide association studies have revealed several common genetic risk variants for ulcerative colitis (UC). However, little is known about the contribution of rare, large effect genetic variants to UC susceptibility. In this study, we performed a deep targeted re-sequencing of 122 genes in Dutch UC patients in order to investigate the contribution of rare variants to the genetic susceptibility to UC. The selection of genes consists of 111 established human UC susceptibility genes and 11 genes that lead to spontaneous colitis when knocked-out in mice. In addition, we sequenced the promoter regions of 45 genes where known variants exert *cis*-eQTL-effects. Targeted pooled re-sequencing was performed on DNA of 790 Dutch UC cases. The Genome of the Netherlands project provided sequence data of 500 healthy controls. After quality control and prioritization based on allele frequency and pathogenicity probability, follow-up genotyping of 171 rare variants was performed on 1021 Dutch UC cases and 1166 Dutch controls. Single-variant association and gene-based analyses identified an association of rare variants in the *MUC2* gene with UC. The associated variants in the Dutch population could not be replicated in a German replication cohort (1026 UC cases, 3532 controls). In conclusion, this study has identified a putative role for *MUC2* on UC susceptibility in the Dutch population and suggests a population-specific contribution of rare variants to UC.

## Introduction

Inflammatory bowel diseases (IBD) are common chronic gastrointestinal inflammatory disorders. The two major forms of IBD are Crohn’s disease (CD) and ulcerative colitis (UC). CD can affect any part of the gastrointestinal tract, while UC is restricted to the colon and the rectum. UC is probably caused by an aberrant immune response against the commensal intestinal flora, influenced by a combination of genetic, microbial and environmental factors, resulting in chronic inflammation of the colonic epithelium. Defects in both innate and adaptive immunity and epithelial barrier function are associated with UC[1].

The genetics of complex diseases has been thoroughly investigated in genome wide association studies (GWAS). These identified thousands of common genetic variants associated with disease susceptibility[2]. GWAS and meta-analyses have identified 200 risk loci in IBD, including 29 risk loci specifically associated with UC. While relevant disease pathways have been identified by GWAS, UC-associated common variants only explain 8.2% of variance in disease onset[3]. Therefore, research looking into the missing heritability in UC is now focused on the contribution of low frequency and rare variants[4,5].

Sequencing studies have revealed that low frequency (minor allele frequency (MAF) between 1% and 5%) and rare (MAF < 1%) genetic variants are more likely to have a deleterious effect on health compared to common variants (MAF > 5%)[6]. Also, population-based studies characterizing detailed genetic variation within a population, like the Genome of The Netherlands (GoNL), have shown that rare genetic variants can be very population-specific[7].

So far, four re-sequencing studies investigating IBD in European populations have been performed[8–11]. Only one of these studies focused on UC[10]. These four studies showed that low frequency and rare protein coding variants in four genes (*NOD2*, *IL23R*, *CARD9* and *BTNL2*) are associated with IBD (p < 1 x 10^−6^). Six additional genes (*IL18RAP*, *CUL2*, *C1orf106*, *PTPN22*, *MUC19* and *RNF186*) are suggestively associated with IBD (p < 0.0001)[8,10,11,9].

Since rare variants are population-specific and only one previous study investigated UC, we aimed to further investigate the contribution of rare, large effect genetic variants to UC susceptibility. We identified a putative role of variants in the *MUC2* gene on UC susceptibility in the Dutch population and suggest a population-specific contribution of rare variants to UC liability.

## Materials and Methods

We performed a targeted resequencing study in 790 UC patients (Phase I) followed by replication of identified variants in an independent Dutch cohort of 1021 UC cases and 1161 Healthy controls (Phase II) and a German cohort consisting of 1026 UC cases and 3532 healthy controls (Phase III).

Pooled targeted deep high-throughput sequencing has been performed of 122 genes: We have selected two groups of target genes for re-sequencing.

**Fig 1:**
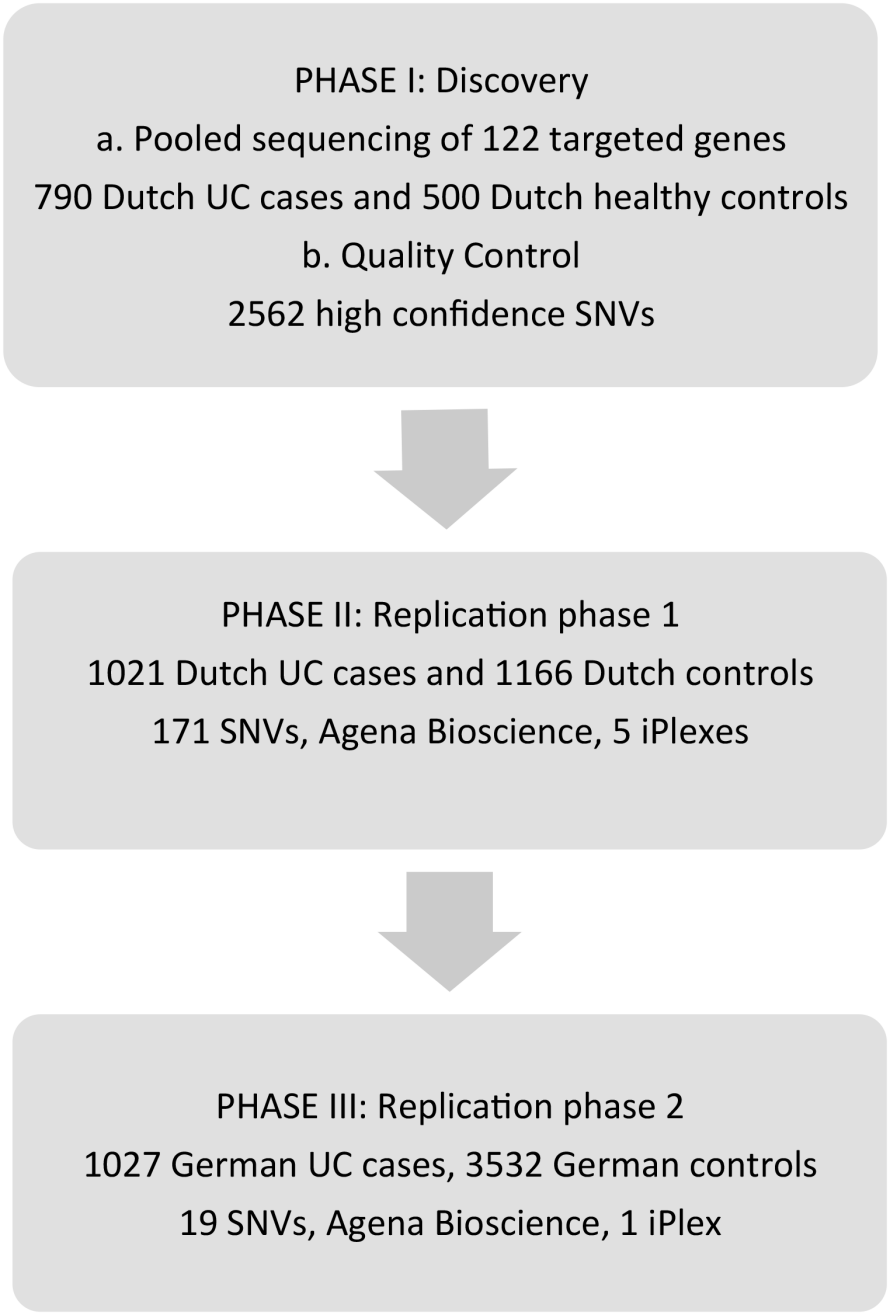
Overview of the screening and replication strategy for rare variants. Phase I: a) targeted re-sequencing of 122 genes was performed in a pooled design of 790 Dutch UC cases. Five hundred healthy individuals sequenced by the Genome of the Netherlands Project were used as a control cohort. After quality control, 2562 high-confidence variants were further prioritized based on allele frequency and likely pathogenicity. In total 188 SNVs were selected for replication phase 1 (Phase II), of which 171 passed the design of five Agena Biosience iPlexes. (http://agenabio.com) b) Phase II: genotyping of 171 variants was performed in 1021 Dutch UC cases and 1166 controls. c) Phase III: after association and gene-based analyses, genotyping of 19 variants was performed in 1026 German UC cases and 3532 healthy German controls.

The first group of genes (n = 111) originates from genomic loci identified through previous GWAS and Immunochip studies conducted by the International IBD Genetics Consortium[12], The second group consisted of genes selected based on the fact that they lead to the development of a spontaneous colitis in knock-out mice (n = 11) [13] (Phase I). (S1 File) In addition to the coding sequence, for 45 of these genes with a known *cis*-eQTL effect (expression Quantitative Trait Locus) we also sequenced the promoter region[14]. We used whole genome sequence data of 500 healthy unrelated Dutch individuals from the Genome of the Netherlands (GoNL) as a control cohort[7]. Follow-up genotyping of identified variants was performed in 1021 Dutch cases and 1166 healthy controls (Phase II) and in independent German cohorts of 1026 UC cases and 3532 healthy controls (Phase III). Fig 1 shows an overview of our analysis strategy (Phases I, II and III).

### Phase I: Discovery

#### Target selection, design and enrichment

In total, for 122 genes, we sequenced all exons including 20 flanking intronic base pairs. In addition, for the genes with a known *cis*-eQTL effect[15], we included 1000 base pairs upstream of the transcription start site in the sequencing design to enable us to identify regulatory variants in the promoter sequence of those genes.

Pooled targeted enrichment of DNA from 790 Dutch UC patients (12 individuals per pool) was performed using a custom-made kit (Agilent HaloPlex). The HaloPlex kit was designed with Agilent’s Sure Design, resulting in coverage of 99.9% of the target sequence (S1 File).

#### Sequencing, read alignment and annotation

Next, after the enrichment, the resulting libraries were sequenced using 100 bp paired-end sequencing on an Illumina HiSeq 2500 machine with 8 barcoded pools per sequence lane. Sequences were aligned using an in house-developed pipeline adapted for pooled sequencing (Genome Build 37, Genome Analysis Toolkit [GATK]). To reduce false-positive SNVs, we performed a second alignment and variant calling with NextGENE software (http://www.softgenetics.com/NextGENe.html). Only variants called by both algorithms were included for further analysis.

Chi-squared and the Fisher-exact tests with R statistical software[7] were used for association analyses. The allele frequency was based on allele counts per Single Nucleotide Variant (SNV). Variants were annotated using SNPeff and SeattleSeq [16,17]. To check for regulatory functions of the variants, the Encyclopedia of DNA Elements (ENCODE)[18] was searched using the UCSC Genome Browser[19].

#### Quality control and variant selection: prioritization of relevant variants

**Fig 2:**
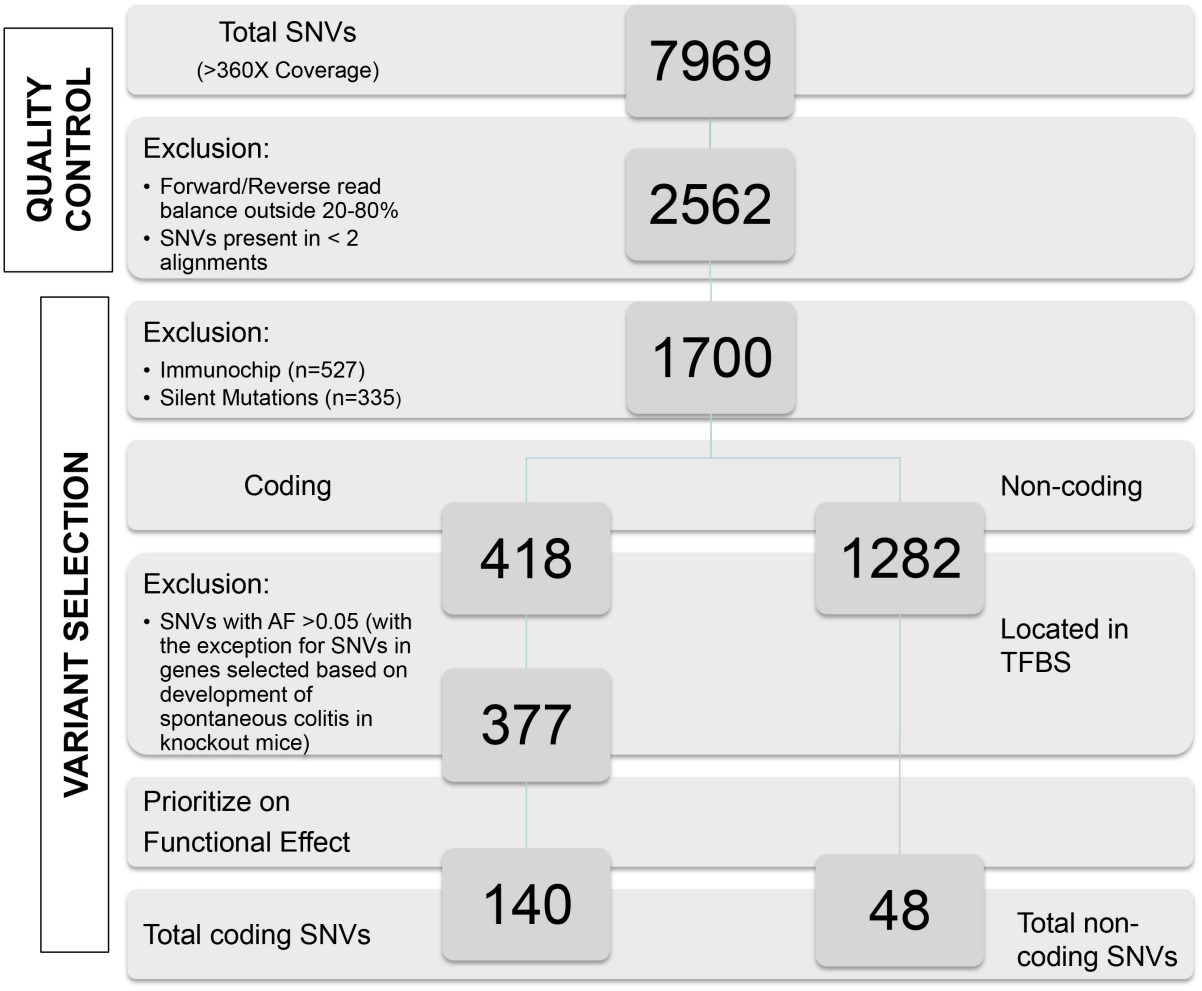
Overview of quality control and prioritization in Phase I. a) After pooled sequencing, a total of 7969 SNVs were detected with a coverage of >360x (12 individuals* 30x coverage). b) All variants called by two alignment strategies were included and filtered using a Forward/Reverse balance between 20–80%. c) Variants previously tested in a large IBD cohort with the Immunochip (n = 527) and silent mutations (n = 335) were excluded. d) We used different strategies to select non-synonymous SNVs (coding), including splice-sites, (n = 418) (d1) and non-coding SNVs (n = 1282) (d2). d1) The coding variants were selected on the basis of allele frequency (AF): known SNVs with an AF > 0.05 were excluded. A different strategy was obtained for genes that are known to lead to spontaneous colitis when in knocked-out mice. In this group of genes we took a more liberal approach in selecting variants for further follow-up and included common variants with predicted functional consequences for follow-up genotyping. Three hundred seventy-seven SNVs remained after this step. d2) To prioritize the non-coding SNVs in regulatory regions, we selected 48 SNVs in a transcription factor binding site (TFBS), based on ENCODE data in the UCSC browser e) Further prioritization was based on damaging effect prediction by Polyphen (damaging effects between 0.8 and 1.0) and/or damaging effect predicted by Sift (n = 112). We included all nonsense variants (n = 6), the variants in splice-sites (n = 4) and variants that were significantly different in AF compared to the AF in GoNL (n = 5). We also included unknown SNVs present in more than one pool (n = 13). f) In total, 140 coding and 48 non-coding rare variants remained after filtering.

As part of our quality control procedure several identified variants were validated by Sanger sequencing (S1 File). An overview of the quality control steps is shown in Fig 2 and described in detail in S1 File.

After quality control, a total of 2562 confidential SNVs remained (S1 Table). To prioritize relevant variants for follow up genotyping, we removed SNVs that had been tested previously in other studies that used the Immunochip genotyping array (n = 527)[12]. Synonymous mutations (n = 335) were removed since they lack functional consequence. Next, we used the following strategies to select non-synonymous SNVs (coding), including splice-sites, (n = 418) as well as non-coding SNVs (n = 1282).

In the coding variant group, we used an allele frequency (AF) threshold of <0.05 for inclusion of variants for follow-up genotyping since common variant (AF > 0.05) analyses within these regions have extensively been performed within the original GWAS and Immunochip based studies[12]. A slightly different strategy was obtained for genes that are known to lead to spontaneous colitis when knocked-out in mice. Here the aim was to study whether genomic variants in these genes exist in humans and whether they are associated with UC susceptibility. In this group of genes we took a more liberal approach in selecting variants for further follow-up and included common variants with predicted functional consequences for follow-up genotyping (Fig 2). After this step, 377 SNVs remained. Further prioritization was based on damaging effect predicted by Polyphen (damaging effects between 0.8 and 1.0) and/or damaging effect predicted by Sift (n = 112). We included all nonsense variants (n = 6), the variants in splice sites (n = 4) and variants that were significantly different in AF compared to the AF in GoNL (n = 5). We also included newly identified variants that were present in multiple pools (n = 13). In total, 140 coding variants remained after this filtering step.

To prioritize the non-coding SNVs in regulatory regions, we selected 48 SNVs in a transcription factor binding site (TFBS), based on ENCODE data in the UCSC browser[19].

In total 188 SNVs were selected for replication phase 1 (Phase II), of which 171 passed the design of five Agena Biosience iPlexes (http://agenabio.com) (S1 Table).

### Phase II: Replication phase 1

Genotyping of 171 SNVs was performed in 1053 independent Dutch UC cases collected as part of the Parelsnoer Institute cohort, and 1170 geographically matched general-population-based Dutch controls with Agena Bioscience iPlex (http://agenabio.com). After quality control (S1 File), the dataset consisted of 1021 UC cases, 1166 healthy controls and 111 SNVs, with a genotype call rate of 98% (S2 Table). Allelic association analysis (χ^2^ test, PLINK v1.07[20]) and permutation (10,000 x) association analysis was done with the Mega-analysis of Rare Variants (MARV) software with a significance cut-off p-value of p<0.05 [9]. EPACTS software was used to perform the gene-based test SKAT-O on 45 genes (all variants with AF<0.05). SKAT-O properly corrects for population substructure. (http://genome.sph.umich.edu/wiki/EPACTS)[9].

In total, 19 variants were selected for replication in an independent cohort (Phase III), including variants with a significant p-value (p< 0.05), singletons replicated in cases in Phase II and SNVs based on the gene-based analysis. SNVs were excluded if the association was in the opposite direction between discovery (Phase I) and replication phase 1 (Phase II).

### Phase III: Replication phase 2

Next, nineteen SNVs were genotyped in 1064 German UC cases and 3576 general-population-based German controls with the iPlex Agena Bioscience system (http://agenabio.com). After quality control (S1 File), the dataset consisted of 1027 UC cases, 3532 healthy controls and 17 SNVs, with a genotype call rate of >99%. Permutation (10,000X) allelic association analysis was performed with the MARV software with a cut-off p-value of p<0.05 [9].

#### Institutional review board approval

Written informed consent was obtained from all participants and the study was approved by the Institutional Review Boards of all individual participating centers:

University Medical Centre Groningen, Groningen, The Netherlands;

Radboud University Nijmegen Medical Centre, Nijmegen, the Netherlands;

Maastricht University Medical Centre, Maastricht, the Netherlands;

Leiden University Medical Centre, Leiden, the Netherlands;

Erasmus University Medical Centre, Rotterdam, the Netherlands;

VU University Medical Centre, Amsterdam, the Netherlands;

University Medical Centre Utrecht, Utrecht, the Netherlands;

Academic Medical Centre, Amsterdam, The Netherlands;

University Medical Centre Schleswig-Holstein, Kiel, Germany.

## Results

Pooled targeted enrichment with Haloplex capturing resulted in coverage of 98%. The mean total number of reads per pool was 36 million, resulting in a mean coverage per pool of 2853x, corresponding with a mean of 238x per individual sample (range 59-450x).

In total, 7969 SNVs were detected with a coverage of >30x per individual. Fifty-two percent of SNVs were known in dbSNP version 137. This fraction is similar to that seen in previous studies [10]. After quality control, a total of 2562 high confidence SNVs remained, resulting in a transition/transversion ratio ti/tv = 2.52 (S1 Table). We confirmed several previously reported rare variants in *IL23R* (rs41313262, rs76418789, rs11209026), *CARD9* (rs141992399, rs200735402) and *JAK2* (rs41316003) (Table 1) [8–11,21]. We excluded these variants from our follow-up because they had already been extensively tested in large cohorts. In all, 877 of the 2562 variants (~35%) were coding variants, and the remainder were located in untranslated regions (n = 110), putative splice sites (n = 8) and intergenic regions (n = 1567) (S1 Table). Ten predicted “loss of function” variants were detected that had not been previously tested in UC GWAS or Immunochip experiments, and these were prioritized for follow-up (Table 2).

**Table 1:** Overview of known rare IBD risk variants.

					Rivas et al			Beaudoin et al			Prescott et al			Hong et al			This study		
					Allele Frequency			Allele Frequency			Allele Frequency			Allele Frequency			Allele Frequency		
SNV	Chr:Position (Hg19)	Gene	Amino Acid Change	cDNA Change	Cases (ICHIP)	Controls (ICHIP)	P	Cases (ICHIP)	Controls (ICHIP)	P	Cases	Controls	P	Cases	Controls	P	Cases	Controls	P
rs41313262^a^	1:67705900	IL23R	p.Val362Ile	c.1084G>A	0.0110	0.0152	1.18 x 10^−5^	0.0012	0.0015	1.2 x 10^−3^	0.0062	0.0139	0.1398	NA	NA	NA	0.0107	0.0210	0.0432
rs76418789^a^	1:67648596	IL23R	p.Gly149Arg	c.445G > A	0.0025	0.0043	3.20 x 10^−4^	0.0034	0.0044	0.0320	0.0016	0.0039	0.8800	0.036	0.068	1.1 x 10^−8^	0.0013	0.0041	0.0040
rs11209026^b^	1:67705958	IL23R	p.Arg381Gln	c.1142G>A	NA	NA	NA	NA	NA	NA	0.0190	0.0570	0.0006	NA	NA	NA	0.0468	0.0750	0.0031
rs141992399^a^	9:139259592	CARD9	NA	c.IVS11+iG>C	0.0024	0.0071	<1. x 10−^16^	0.0003	0.0007	1.5 x 10^−11^	NA	NA	NA	NA	NA	NA	0.0025	0.0070	0.1199
rs200735402^c^	9:139265120	CARD9	p.Glu221Lys	c.661G>A	NA	NA	NA	NA	NA	NA	NA	NA	NA	0.001	0.011	0.0001	NA	NA	NA
rs41316003^d^	9:5126343	JAK2	p.Arg1063His	c.3188G>A	NA	NA	NA	0.00034*	0.00058*	0.0150	NA	NA	NA	NA	NA	NA	0.0190	0.0120	0.2027

This table provides an overview of known rare IBD variants, based on literature. Exclusively, the genes included in our UC study design are displayed. The allele frequencies and p-values of combined analyses of the variants in the different studies (Rivas et al(9), Beaudoin et al(7), Prescott et al(10), Hong et al(21), and our study (Discovery, Phase I) are shown.

^a^ identified by Rivas et al(9).

^b^ identified by Momozawa et al(8).

^c^ identified by Hong et al(21), not replicated in the other populations.

^d^ identified by Beaudoin et al(10) in the follow-up phase, but not tested for replication on the Immunochip.

SNV: single nucleotide variant; Chr: chromosome;, ICHIP: Immunochip, P: P-value, NA not applicable

**Table 2:** Predicted loss of function variants identified by pooled sequencing (Phase I), and genotyped in replication phase 1 (Phase II).

						Discovery (Phase I)			Replication phase 1 (Phase II)			
						Allele Frequency			Allele Frequency			
SNV	Chr:Position (Hg19)	Gene	Amino Acid Change	cDNA Change	Exonic function	Cases	Controls (GoNL)	P_FISHER	Cases	Controls	P_CHISQ	P_10,000perm
-	2:25064537	*ADCY3*	NA	c.957-1G>T	SPLICE_SITE_ACCEPTOR	0.0006	NA*	NA	fail QC	fail QC	fail QC	NA
rs150302537	2:28532947	*BRE*	NA	c.1089-2A>C	SPLICE_SITE_ACCEPTOR	0.0038	0.0020	0.7186	0.0020	0.0043	0.1783	0.0880
-	1:67702486	*IL23R*	NA	c.1045+1G>T	SPLICE_SITE_DONOR	0.0006	NA*	NA	NA*	0.0004	0.3517	0.1869
-	20:62369002	*LIME1*	NA	c.98+2T>C	SPLICE_SITE_DONOR	0.0006	NA*	NA	NA*	0.0009	0.1745	0.0858
rs142690032	3:49721812	*MST1*	p.Arg651*	c.1951C>T	STOP_GAINED	0.0107	0.0080	0.5427	0.0182	0.0139	0.2502	0.1502
rs147438510	7:36561695	*AOAH*	p.Gly517*	c.1549G>T	STOP_GAINED	0.0044	0.0040	1.0000	0.0025	0.0022	0.8398	0.4118
-	11:64111929	*CCDC88B*	p.Trp639*	c.1916G>A	STOP_GAINED	0.0006	NA*	NA	NA*	NA*	NA	NA
-	12:12588642	*LOH12CR1*	p.Arg95*	c.283C>T	STOP_GAINED	0.0006	NA*	NA	fail QC	fail QC	fail QC	NA
-	20:62328835	*TNFRSF6B*	p.Cys193*	c.579C>A	STOP_GAINED	0.0006	NA*	NA	NA*	NA*	NA	NA
-	22:30415593	*MTMR3*	p.Glu649*	c.1945G>T	STOP_GAINED	0.0013	NA*	NA	NA*	NA*	NA	NA

Pooled sequencing identified 10 predicted loss of function variants, shown in this table. The exonic function is predicted based on SNPeff. Allele frequencies of the discovery (Phase I) and the replication phase 1 (Phase II) are provided.

* no carriers detected

SNV: single nucleotide variant; Chr: chromosome; GoNL: Genome of the Netherlands; P_CHISQ: p-value of chi-squared; P_Fisher: p-value of fisher exact test, P_10,000perm: p-value of 10,000 permutations; fail QC: variants fail the quality control; NA: not applicable.

In total, 188 SNVs were selected for follow-up genotyping, of which 171 passed the design of the Agena Bioscience iPlex (Phase II). After quality control 111 SNVs remained. The relatively low number of replicated SNVs results from the stringent cut-off threshold to exclude false positives. For 30 of the 111 rare SNVs, we could not identify additional carriers in either cases or controls. For half of the variants, we detected a discrepancy in the direction of the AF between cases and controls in the discovery (Phase I) and replication phase 1 (Phase II). For one singleton variant, we detected one additional carrier in the cases. For the SNVs located in a TFBS, we detected nine additional carriers, but no significant differences in AF between the cases and controls in the replication phase 1 (Phase II, (S2 Table).

Single marker permutation (10,000x) allelic association analysis, performed with the Mega-analysis of Rare Variants (MARV) software, detected eight SNVs (P < 0.05) with a significant difference in AF between cases and controls[9]. Four of these SNVs were located in the coding region of *MUC2*. The other four SNVs consisted of one stop-gain variant located in *CCDC88B*, two damaging coding variants in *RFTN2* and *MMEL1* and one variant in a TFBS in the promoter region of the *PMCA* gene (Table 3). Gene-based analysis with SKAT-O resulted in nine variants in the *MUC2* gene with a significant p-value of 9.2 x 10^−5^ (threshold p = 0.0011 after Bonferroni correction).

**Table 3:** Significant SNVs in Replication phase 1 (Phase II) and replication phase 2 (Phase III).

					Discovery (Phase I)			Replication phase 1 (Phase II)					Replication phase 2 (Phase III)					Exac
					Allele Frequency			Allele Frequency					Allele Frequency					
SNV	Chr:Position (Hg19)	GENE	Amino Acid Change	cDNA Change	Cases	Controls	P_FISHER	Cases	Controls	P_CHISQ	OR	P_10,000 perm	Cases	Controls	P_CHISQ	OR	P_10,000 perm	Euro_freq
rs147664779	11:1083557	*MUC2*	p.Arg743Trp	c.2227C>T	0.0013	0.0000	NA	0.0070	0.0009	0.0009	8.1940	0.0003	fail QC	fail QC	fail QC	NA	NA	0.0006
rs41376152	11:1094761	*MUC2*	p.Thr1946Asn	c.5837C>A	0.0316	0.0240	0.2783	0.0657	0.0361	1.15E-05	1.8790	0.0057	0.0274	0.0274	0.3853	0.8781	0.1887	0.0289
rs4400498*	9:139305007	*PMPCA*	NA	c.-158G>A	0.0923	0.2390	<0.0001	0.0093	0.0180	0.0166	0.5108	0.0065	0.3280	0.3126	0.1811	1.073	0.0863	NA
rs2856111	11:1075747	*MUC2*	p.Leu58Pro	c.173T>C	0.1517	0.1320	0.1857	0.1445	0.1239	0.0487	1.1940	0.0321	NA	NA	NA	NA	NA	0.1346
rs149995388	2:198482574	*RFTN2*	p.Ser334Arg	c.1000A>C	0.0088	0.0070	0.8210	0.0079	0.0039	0.0790	2.0510	0.0339	NA	NA	NA	NA	NA	0.0047
rs150660153	1:2535397	*MMEL1*	p.Glu323Gln	c.967G>C	0.0019	0.0020	1.0000	0.0010	0.0035	0.0906	0.2850	0.0442	NA	NA	NA	NA	NA	0.0027
rs41386154	11:1097749	*MUC2*	p.Asn2277Thr	c.6830A>C	0.0126	0.0070	0.0325	0.0066	0.0030	0.0842	2.2050	0.0485	0.0024	0.0024	0.2441	0.5726	0.13	0.0021
rs144037797	11:64117106	*CCDC88B*	p.Thr943Ile	c.2828C>T	0.0278	0.0320	0.5513	0.0223	0.0314	0.0664	0.7041	0.0492	NA	NA	NA	NA	NA	0.0344

Table 3 shows all significant associated SNVs in replication phase 1 (Phase II) and replication phase 2 (Phase III). Phase I: 790 UC cases, 500 GoNL controls; Phase II: 1021 UC cases, 1166 healthy controls; Phase III: 1026 German UC cases, 3532 healthy German controls. Besides, the allele frequencies of the Exac database or shown. The *MUC2* gene is selected based on the fact that this gene leads to the development of a spontaneous colitis in knock-out mice. Fur *MUC2* we took a more liberal approach in selecting variants and included common variants with predicted functional consequences for follow up genotyping.

* Follow-up genotyping of rs4400498 in the *PMCA* gene had a 10-times difference in AF in the replication phase 1 (Phase II) compared to the replication phase 2 (Phase III). This is probably due an artefact in phase II.

SNV: single nucleotide variant; Chr: chromosome; UC: Ulcerative Colitis; freq: allele frequency; GoNL: Genome of the Netherlands; P_CHISQ: p-value of chi-squared; OR: Odds Ratio; P_10,000perm: p-value of 10,000 permutations; NA: not applicable, Euro_freq: allele frequencies of european (non-Finnish) population in Exac database (http://exac.broadinstitute.org)

In total, 19 variants were selected for replication phase 2 (Phase III). After quality control, 17 variants remained, and none of the variants were associated with UC in the German cohort (Phase III).

## Discussion

In this large Dutch sequencing study, we investigated the contribution of rare variants to the genetic susceptibility of UC. We identified a supposed role for the *MUC2* gene on UC susceptibility in the Dutch population, suggesting a population-specific contribution of rare variants to UC susceptibility. What distinguishes our study from previous re-sequencing studies is that we include 11 genes that are known to lead to spontaneous colitis when knocked-out in mice[13]. Moreover, we include the promoter regions of genes with a known *cis*-eQTL effect. We have sequenced 122 genes in 790 Dutch UC patients, using a targeted pooled sequencing approach. After prioritization of variants with a pathogenic probability, extensive follow-up genotyping in ~1000 additional Dutch UC cases and ~1200 healthy Dutch controls revealed an association of variants in the *MUC2* gene with UC in the Dutch population. This association was not replicated in an independent German cohort. We also confirmed known rare variants in the *IL23R* (rs41313262, rs76418789, rs11209026), *CARD9* (rs141992399, rs200735402) and *JAK2* (rs41316003) genes, most with similar AFs to those reported in other studies (Table 1).

Pooled sequencing has proven to be a highly cost-effective method for screening large populations. Therefore, it has been used in several re-sequencing studies in IBD [9–11,21]. A major problem of sequencing studies is the relative high rate of false-positive SNVs. The recommended approach to minimize the high false-positive rate is very deep sequencing (100x per individual) of a large population with geographically matched individuals [22]. In this study, we used the largest Dutch UC cohort available for discovery (Phase I) and replication phase 1 (Phase II). Target enrichment was performed with HaloPlex capturing, in which genomic DNA is fragmented by restriction enzyme digestion and circularized by hybridization to probes. Compared to hybrid capture methods, HaloPlex is relatively quick and inexpensive. It also requires a smaller amount of DNA and has a higher fraction of sequence reads in our region of interest [23]. However, because of the fragmentation with restriction enzymes instead of random fragmentation, it is impossible to exclude duplicate reads in the alignment in order to reduce sequencing artefacts. Therefore, we used the presence of the SNVs in both forward and reverse sequencing reads as a quality control filter, which substantially reduced the number of false positives. Since this output cannot be deduced from our standard bioinformatics GATK-pipeline, we did additional alignment and variant calling using the NextGene Software. After an extensive, stringent quality control with the additional alignment, ~2500 highly confident variants remained with a minimal coverage of >59x and with a transition/transversion ratio ti/tv = 2.52, indicative of a relatively high true-positive rate for our dataset[10,9].

Single marker association and gene-based analyses (p-value = 9.2 x 10^−5^) showed an association of the *MUC2* gene with UC in the Dutch population (Table 3). *MUC2* was selected because it induces spontaneous colitis when knocked out in mice[1,13,24]. The *MUC2* gene encodes a member of the mucin protein family and is the major mucin secreted in the large intestine. The colonic mucus layer plays a critical role in intestinal homeostasis by limiting contact between luminal bacteria and the mucosal immune system. A defective mucosal barrier is a key feature of active UC[25,26]; patients with UC present with a reduction of goblet cells, decreased glycosylation of mucins, and absence of *MUC2* expression in goblet cells in the affected colon mucosa. Altogether, this functional evidence supports *MUC2* as a candidate gene for UC pathogenesis.

*MUC2* has not been previously identified as an UC-associated gene. A previous small candidate-approach genetic association study did not show an association of *MUC2* with UC[3]. Furthermore, *MUC2* has never been associated with UC in GWAS studies or meta-analyses; the Immunochip contains just two *MUC2* SNPs and only a few were present on previous GWAS platforms (Illumina HumanHap550). The reason for this could be the difficulty of designing specific probes because of the homology of the *MUC2* gene with other members of the mucin protein family (*MUC5AC*, *MUC5B*, *MUC6* and *MUC19*). This strong homology could be a source of problems in the alignment of sequencing reads, thereby introducing false positive SNVs. However, we were able to validate our variants using Agena Bioscience assays, which were highly specific for *MUC2* as demonstrated by blasting of our sequences in the UCSC genome browser (http://genome.ucsc.edu). Blast output and a clusterplot of *MUC2* is shown in the S1 file. *MUC2* is a very large gene. The exonic sequence contains 49 exons and the entire *MUC2* gene product has more than 5100 amino acids in its commonest allelic form. The size of the gene makes it more likely to detect mutations.

While our association of *MUC2* in the Dutch UC population could not be replicated in a German cohort, this might be because our associated SNVs are population specific or because of a lack of power. Recently, the first trans-ancestry association study in IBD was performed in a cohort of 86,640 European individuals and 9,846 individuals of East Asian, Indian or Iranian descent [3]. The majority of the loci found, based on common SNPs with a MAF >5%, were shared between different ancestry groups. However, this study also found genetic heterogeneity between divergent populations at several established risk loci driven by difference in allele frequency (*NOD2*) or effect size (*TNFSF15* and *ATG16L1*), or a combination of these factors (*IL23R* and *IRGM*). Rare variants are even more likely to be specific to a particular population, as was demonstrated by a recent sequencing study in a Korean IBD population [21]. Table 1 shows that allele frequencies for a rare variant in *IL23R* (rs76418789) differ strongly among populations, even between closely related UK populations [11] in Prescott’s study and the large population used in the Rivas and Beaudoin studies (NIDDK consortium (North America), Australia, Italian, Dutch, Swedish, German, UK population) [10,9]. The Korean study shows a 10x higher allele frequency compared to European populations [21]. These differences in MAF between populations, even in ancestrally close populations, could explain the lack of replication between our Dutch and German cohorts. There could also simply be a lack of power to detect association in our replication phase 2 (Phase III, Table 3). For example, the *CARD9* splice-site (rs141992399) has the same allele frequency in the large population of the Rivas paper (28,000 patients and 17,570 healthy controls) as in our study, but our p-value is much higher (Table 1), which underlines the importance of well-powered studies to detect significant rare variants.

Large whole genome sequencing (WGS) and whole exome sequencing (WES) studies in IBD are in progress. Although we identified potential variants in TFBS, none of them were statistically significant in replication phase 1 (Phase II). Thus the WGS and WES studies will increase the power to explore the non-coding part of the genome and the association of the *MUC2* region to UC in different populations.

## Conclusions

Identifying associations of rare variants in complex diseases remains challenging, and the approach of re-sequencing known genes might not be the key to resolving the missing heritability in complex diseases like UC. The power of rare variants could be better captured in the regulatory, non-coding part of the genome by sequencing the whole genome or, specifically, the enhancer regions. Another option is to select genes based on pathway analyses or candidate genes, or to use specific phenotypic populations (like early onset IBD or family based studies). If the eventual goal is individual risk-scores for disease development, genomic interpretation of the non-coding part of the genome is crucial. For this, large well powered WGS and WES studies are necessary to give a realistic view of the role of rare variants in complex disease.

## Supporting Information

S1 File includes supplementary list of 122 selected genes, Supplementary Methods and Supplementary Blasting and Cluster plot example. (DOCX)

S1 Table Discovery phase (Phase I): complete list of 2562 SNVs after quality control. Pooled re-sequencing of 122 UC genes in 790 Dutch UC patients resulted in 7969 SNVs. After quality control (see material and methods, Fig 2) 2562 SNVs remained. This list provides an overview of all 2562 SNVs including association analysis with the control cohort, annotation and selection of variants for follow-up. Association analysis was done with the Chi-squared and the Fisher-exact tests with R statistical software. The allele frequency was based on allele counts per SNV. Variants were annotated using SNPeff and SeattleSeq. To check for regulatory functions of the variants, the Encyclopedia of DNA Elements (ENCODE) was searched using the UCSC Genome Browser. **Chromosome** = chromosome number (Hg 19), **Position** = base pair position (Hg 19), **Chr:position** = combined chromosome and position (Hg 19), **Nr_pools** = number of pools (of 12 patients) in which variant is detected, **SNV** = rs-id if available from dbSNP137, **refAllele** = reference allele, **altAllele** = alternative allele, **UC_freq** = allele frequency detected in 790 UC patients, **Controls_GoNL_Freq** = allele frequency detected in 500 healthy controls of the Genome of the Netherlands cohort(GoNL), **CHISQ** = p-value after Chi-squared test, **FISHER** = p-value after Fisher-exact test, **Wash_EA_AF** = allele frequency based on European population in Exome Variant Server (http://evs.gs.washington.edu/EVS/), **1000G_EUR_AF** = allele frequency based on European population in 1000 genomes (http://www.1000genomes.org), **ExAC** = allele frequency based on Exome Aggregation Consortium (http://exac.broadinstitute.org), **HGVS.c** = Variant using Human Genome Variation Society notation (DNA level), **HGVS.p** = If variant is coding, this field describes the variant using Human Genome Variation Society notation(Protein level), **SnpEff_effect =** Effect of this variant based on SnpEff, **SnpEff_gene_biotype =** This field is 'CODING' if any transcript of the gene is marked as protein coding, **SnpEff_gene_name** = name of the Gene, **Selection group =** genes selected in UC genes or genest hat lead to spontaneous colitis when knocked-out in mice. **DNASE1 =** DNase I hypersensitive sites from ENCODE, **HISTONE =** histone modification from ENCODE, **POLYMERASE** = polymerase subunits from ENCODE, **TFBS** = Transcription Factor Binding Sites from ENCODE, **DNASE1_CELLTYPES** = DNase I hypersensitive sites specific cell types from ENCODE, **HISTONE_CELLTYPES** = histone modification specific cell types from ENCODE, **POLYMERASE_CELLTYPES =** polymerase specific cell types from ENCODE, **TFBS_CELLTYPES** = Transcription Factor Binding Sites specific cell types from ENCODE, **PolyPhen** = polymorphism phenotyping, used to predict functional effect of human missense variant, in this study the damaging effect cut-off is between 0.8–1.0. **ClinicalAssociation** = link with known clinical association, **SIFT** = predicts whether an amino acid substitution affects protein function, **SNP_on_ICHIP** = SNP already tested on Immunochip, **Refseq** = annotation based on Reference sequence database (http://www.ncbi.nlm.nih.gov/refseq/), **Imputed_SNV_ICHIP =** variant is imputed in Immunochip dataset, using GoNL data **Selected_follow-up** = variants selected for follow-up (Phase II), 140 coding variants, 48 variants based on location in Transcription Factor Binding Sites.
(XLSX)

S2 Table Replication phase 1 (Phase II): complete list of 111 SNVs after quality control. Follow-up genotyping of 171 SNVs (after quality control) in an additional Dutch cohort (funded by the Parelsnoer Institute) consisted of 1021 UC cases, 1166 healthy controls and 111 SNVs, with a genotype call rate of 98%. Allelic association analysis (χ^2^ test, PLINK v1.07) and permutation (10,000X) association analysis was performed with the Mega-analysis of Rare Variants (MARV) software, significance cut-off p-value of <0.05. A Gene-Based analysis was performed with EPACTS-software, 45 genes (all variants with AF < 0.05) were tested with the SKAT-O test. (http://genome.sph.umich.edu/wiki/EPACTS). **OR** = Odds Ratio, **Zstat_10,000perm** = Z-statistic (which is compared to a reference standard normal distribution) after 10,000 permutations, **P_10,000perm =** p-value after 10,000 permutations (MARV software).
(XLSX)

## Abbreviations

UC: Ulcerative Colitis
CD: Crohn’s disease
IBD: Inflammatory Bowel Disease
GoNL: Genome of the Netherlands
SNV: Single Nucleotide Variant
AF: Allele Frequency
MAF: Minor Allele Frequency
GWAS: Genome Wide Association Scan
eQTL: expression Quantitative Trait Locus
TFBS: Transcription Factor Binding Site
GATK: The Genome Analysis Toolkit
WGS: Whole Genome Sequencing
WES: Whole Exome Sequencing
ENCODE: The Encyclopedia of DNA Elements
NGS: Next Generation Sequencing
F/R: Forward/Reverse
PCR: Polymerase Chain Reaction
PSI: Parelsnoer Institute
