# Supplementary material for: Pooled Resequencing of 122 Ulcerative Colitis Genes in a Large Dutch Cohort Suggests Population-Specific Associations of Rare Variants in MUC2
Source: PLoS One. 2016 Aug 4;11(8):e0159609. doi: 10.1371/journal.pone.0159609 (PMC4973970; doi:10.1371/journal.pone.0159609)
Supplement: S1 File — (DOCX) [file pone.0159609.s001.docx]

**Supplementary Information**

**Pooled resequencing of 122 ulcerative colitis genes in a large Dutch population suggests population-specific association of rare variants in MUC2**

**Table of Contents**

**Supplementary List- List with 122 selected Genes…………………………………3**

**Supplementary Methods.………...……………………………………………………...4**

**Supplementary Blasting and Cluster plot example…………………………………8**

**Supplementary References…………….....………………………………………….....9**

**List of 122 UC genes: 111 UC genes**[1] **identified through GWAS and 11 genes known to lead to spontaneous colitis when knocked-out in mice (Phase I)**[2]

| \| \| 1 \| RFTN2 \|  \| 43 \| VDR \|  \| 85 \| IRGM \| \| --- \| --- \| --- \| --- \| --- \| --- \| --- \| --- \| \| 2 \| PLCL1 \|  \| 44 \| GPR183 \|  \| 86 \| IL12B \| \| 3 \| ITIH4 \|  \| 45 \| GPR18 \|  \| 87 \| TNFAIP3 \| \| 4 \| MANBA \|  \| 46 \| CRTC3 \|  \| 88 \| JAK2 \| \| 5 \| JRKL \|  \| 47 \| SMAD7 \|  \| 89 \| CARD9 \| \| 6 \| MAML2 \|  \| 48 \| CD226 \|  \| 90 \| CCDC88B \| \| 7 \| TRAF3IP2 \|  \| 49 \| COMMD1 \|  \| 91 \| IFNG \| \| 8 \| SMURF1 \|  \| 50 \| AOAH \|  \| 92 \| GPR65 \| \| 9 \| PRKCB \|  \| 51 \| RUNX3 \|  \| 93 \| GALC \| \| 10 \| SLC9A3 \|  \| 52 \| MUC2 \|  \| 94 \| STAT3 \| \| 11 \| GNA12 \|  \| 53 \| TLR5 \|  \| 95 \| TYK2 \| \| 12 \| DLD \|  \| 54 \| C1GALT1 \|  \| 96 \| CEBPG \| \| 13 \| IRF5 \|  \| 55 \| XBP1 \|  \| 97 \| TNFRSF6B \| \| 14 \| ZFP90 \|  \| 56 \| IKBKG \|  \| 98 \| ICOSLG \| \| 15 \| IRF1 \|  \| 57 \| WAS \|  \| 99 \| LIF \| \| 16 \| SLC22A4 \|  \| 58 \| GPX1 \|  \| 100 \| OSM \| \| 17 \| PDLIM4 \|  \| 59 \| GPX2 \|  \| 101 \| NDUFAF1 \| \| 18 \| SLC22A5 \|  \| 60 \| TNFRSF14 \|  \| 102 \| KIR2DL1 \| \| 19 \| CREM \|  \| 61 \| CARD11 \|  \| 103 \| DNMT3B \| \| 20 \| NK2 homeobox 3 \| \| 62 \| FAM55A \|  \| 104 \| MMEL1 \| \| 21 \| TNNI2 \|  \| 63 \| FAM55D \|  \| 105 \| TNPO3 \| \| 22 \| LSP1 \|  \| 64 \| ADA \|  \| 106 \| CD244 \| \| 23 \| ORMDL3 \|  \| 65 \| HNF4A \|  \| 107 \| SLC11A1 \| \| 24 \| PRKCD \|  \| 66 \| TNFRSF9 \|  \| 108 \| PNKD \| \| 25 \| NFKB1 \|  \| 67 \| IL23R \|  \| 109 \| ARPC2 \| \| 26 \| ITGAL \|  \| 68 \| CD48 \|  \| 110 \| TMBIM1 \| \| 27 \| CALM3 \|  \| 69 \| C1ORF53 \|  \| 111 \| CXCL5 \| \| 28 \| TNFRSF18 \|  \| 70 \| KIF21B \|  \| 112 \| PMPCA \| \| 29 \| TNFRSF4 \|  \| 71 \| IL10 \|  \| 113 \| SDCCAG3 \| \| 30 \| RORC \|  \| 72 \| ADCY3 \|  \| 114 \| INPP5E \| \| 31 \| FOSL2 \|  \| 73 \| GPR35 \|  \| 115 \| GSDMA \| \| 32 \| BRE \|  \| 74 \| MST1 \|  \| 116 \| LIME1 \| \| 33 \| SPRED2 \|  \| 75 \| PFKFB4 \|  \| 117 \| SLC2A4RG \| \| 34 \| IFIH1 \|  \| 76 \| IL2 \|  \| 118 \| ZGPAT \| \| 35 \| STAT1 \|  \| 77 \| IL21 \|  \| 119 \| UBE2L3 \| \| 36 \| STAT4 \|  \| 78 \| PTGER4 \|  \| 120 \| RIMBP3 \| \| 37 \| DOK3 \|  \| 79 \| IL13 \|  \| 121 \| CCDC116 \| \| 38 \| NFIL3 \|  \| 80 \| CSF2 \|  \| 122 \| MTMR3 \| \| 39 \| CNTF \|  \| 81 \| IL4 \|  \|  \|  \| \| 40 \| LPXN \|  \| 82 \| IL3 \|  \|  \|  \| \| 41 \| CD6 \|  \| 83 \| IL5 \|  \|  \|  \| \| 42 \| LOH12CR1 \|  \| 84 \| ACSL6 \|  \|  \|  \| \|  \| \| --- \| --- \| --- \| --- \| --- \| --- \| --- \| --- \| --- \| --- \| --- \| --- \| --- \| --- \| --- \| --- \| --- \| --- \| --- \| --- \| --- \| --- \| --- \| --- \| --- \| --- \| --- \| --- \| --- \| --- \| --- \| --- \| --- \| --- \| --- \| --- \| --- \| --- \| --- \| --- \| --- \| --- \| --- \| --- \| --- \| --- \| --- \| --- \| --- \| --- \| --- \| --- \| --- \| --- \| --- \| --- \| --- \| --- \| --- \| --- \| --- \| --- \| --- \| --- \| --- \| --- \| --- \| --- \| --- \| --- \| --- \| --- \| --- \| --- \| --- \| --- \| --- \| --- \| --- \| --- \| --- \| --- \| --- \| --- \| --- \| --- \| --- \| --- \| --- \| --- \| --- \| --- \| --- \| --- \| --- \| --- \| --- \| --- \| --- \| --- \| --- \| --- \| --- \| --- \| --- \| --- \| --- \| --- \| --- \| --- \| --- \| --- \| --- \| --- \| --- \| --- \| --- \| --- \| --- \| --- \| --- \| --- \| --- \| --- \| --- \| --- \| --- \| --- \| --- \| --- \| --- \| --- \| --- \| --- \| --- \| --- \| --- \| --- \| --- \| --- \| --- \| --- \| --- \| --- \| --- \| --- \| --- \| --- \| --- \| --- \| --- \| --- \| --- \| --- \| --- \| --- \| --- \| --- \| --- \| --- \| --- \| --- \| --- \| --- \| --- \| --- \| --- \| --- \| --- \| --- \| --- \| --- \| --- \| --- \| --- \| --- \| --- \| --- \| --- \| --- \| --- \| --- \| --- \| --- \| --- \| --- \| --- \| --- \| --- \| --- \| --- \| --- \| --- \| --- \| --- \| --- \| --- \| --- \| --- \| --- \| --- \| --- \| --- \| --- \| --- \| --- \| --- \| --- \| --- \| --- \| --- \| --- \| --- \| --- \| --- \| --- \| --- \| --- \| --- \| --- \| --- \| --- \| --- \| --- \| --- \| --- \| --- \| --- \| --- \| --- \| --- \| --- \| --- \| --- \| --- \| --- \| --- \| --- \| --- \| --- \| --- \| --- \| --- \| --- \| --- \| --- \| --- \| --- \| --- \| --- \| --- \| --- \| --- \| --- \| --- \| --- \| --- \| --- \| --- \| --- \| --- \| --- \| --- \| --- \| --- \| --- \| --- \| --- \| --- \| --- \| --- \| --- \| --- \| --- \| --- \| --- \| --- \| --- \| --- \| --- \| --- \| --- \| --- \| --- \| --- \| --- \| --- \| --- \| --- \| --- \| --- \| --- \| --- \| --- \| --- \| --- \| --- \| --- \| --- \| --- \| --- \| --- \| --- \| --- \| --- \| --- \| --- \| --- \| --- \| --- \| --- \| --- \| --- \| --- \| --- \| --- \| --- \| --- \| --- \| --- \| --- \| --- \| --- \| --- \| --- \| --- \| --- \| --- \| --- \| --- \| --- \| --- \| --- \| --- \| --- \| --- \| --- \| --- \| |
| --- | --- | --- | --- | --- | --- | --- | --- | --- | --- | --- | --- | --- | --- | --- | --- | --- | --- | --- | --- | --- | --- | --- | --- | --- | --- | --- | --- | --- | --- | --- | --- | --- | --- | --- | --- | --- | --- | --- | --- | --- | --- | --- | --- | --- | --- | --- | --- | --- | --- | --- | --- | --- | --- | --- | --- | --- | --- | --- | --- | --- | --- | --- | --- | --- | --- | --- | --- | --- | --- | --- | --- | --- | --- | --- | --- | --- | --- | --- | --- | --- | --- | --- | --- | --- | --- | --- | --- | --- | --- | --- | --- | --- | --- | --- | --- | --- | --- | --- | --- | --- | --- | --- | --- | --- | --- | --- | --- | --- | --- | --- | --- | --- | --- | --- | --- | --- | --- | --- | --- | --- | --- | --- | --- | --- | --- | --- | --- | --- | --- | --- | --- | --- | --- | --- | --- | --- | --- | --- | --- | --- | --- | --- | --- | --- | --- | --- | --- | --- | --- | --- | --- | --- | --- | --- | --- | --- | --- | --- | --- | --- | --- | --- | --- | --- | --- | --- | --- | --- | --- | --- | --- | --- | --- | --- | --- | --- | --- | --- | --- | --- | --- | --- | --- | --- | --- | --- | --- | --- | --- | --- | --- | --- | --- | --- | --- | --- | --- | --- | --- | --- | --- | --- | --- | --- | --- | --- | --- | --- | --- | --- | --- | --- | --- | --- | --- | --- | --- | --- | --- | --- | --- | --- | --- | --- | --- | --- | --- | --- | --- | --- | --- | --- | --- | --- | --- | --- | --- | --- | --- | --- | --- | --- | --- | --- | --- | --- | --- | --- | --- | --- | --- | --- | --- | --- | --- | --- | --- | --- | --- | --- | --- | --- | --- | --- | --- | --- | --- | --- | --- | --- | --- | --- | --- | --- | --- | --- | --- | --- | --- | --- | --- | --- | --- | --- | --- | --- | --- | --- | --- | --- | --- | --- | --- | --- | --- | --- | --- | --- | --- | --- | --- | --- | --- | --- | --- | --- | --- | --- | --- | --- | --- | --- | --- | --- | --- | --- | --- | --- | --- | --- | --- | --- | --- | --- | --- | --- | --- | --- | --- | --- | --- | --- | --- | --- | --- | --- | --- | --- |
|  |

**Supplementary methods**

**Ethical approval.**

The recruitment of study subjects was approved by the ethics committees or institutional review boards of all individual participating centers or countries. Written informed consent was obtained from all study participants

**Phase I: Discovery**

**Target selection, subjects, sample preparation and target enrichment**

*Target selection* 111 UC genes were included with association p<10^-7^ and IBD shared genes with UC association p<10^-5^, based on the associated UC and IBD loci of the IIBDGC Immunochip study (133 loci, 184 genes) [1]. We used the prioritization of genes from the loci as described before [1]. We included 11 genes known to lead to spontaneous colitis when knocked-out in mice that might genetically be implicated in UC [2]. In total 122 genes were included.

*Subjects* Patient DNA came from a cohort of 790 Dutch UC patients and was collected in the outpatient IBD clinics of Radboud University of Nijmegen (n=207), Leiden University Medical Center (n=179) and the University Medical Center Groningen (n=404).

*Sample preparation* Genomic DNA samples were purified (using glycoblue (Lot# 0912014, Ambion, USA) and 2-propanol), normalized and measured by spectrophotometry (Nanodrop 8000, Spectrophotometer UV-VIS, Thermo Scientific, USA). DNA samples with an OD 260/280 ratio below 1.7 or above 2.1 were excluded. To guarantee good quality DNA, we verified DNA integrity by agarose gel electrophoresis. DNA samples with smearing below 2.5 kb were excluded. In this quality control step, 128 DNA samples were excluded leaving 790 patients in the cohort. In total 65 pools were composed of 12 DNA samples of equimolar amount using the Janus Robot (PerkinElmer, USA**).** 1 pool of 10 patients was used as a pilot study, which was included in the analyses. Final concentrations of the pools were measured in triplo with the Labchip GX (PerkinElmer, USA).

*Targeted enrichment* Targeted enrichment was performed by the use of a custom-made kit (HaloPlex 1-500 kb, ILMFST Box 1, 96 reactions, #5190-5385, Design ID:01801-1342778020, Agilent Technologies, USA). The HaloPlex design for the 122 genes was made with Agilent’s Sure Design resulting in coverage of 99.9% of the target sequence.

**Quality control**

In order to reduce false-positive SNVs we performed several quality control steps. We used strict coverage criteria > 360 times (12 individuals X 30x coverage) and defined cut-off values after a validation experiment (a detailed description of the validation experiment is described in the next paragraph). Next, we had Immunochip genotyping available for approximately 50% of the patients that were sequenced in the discovery part of the study. We performed imputation of these 437 samples using the population matched Genome of The Netherlands as a reference panel[3]. (An overview of the imputation steps is described in the next paragraph) We then checked whether identified SNVs through sequencing were also identified in the same patients by imputed Immunochip data. If this was the case we considered this as added evidence that the identified rare variants were correctly genotyped and could be selected for follow up genotyping. After these quality control steps, a total of 2562 high confidence SNVs remained.

*Validation experiment* We performed a validation experiment using Sanger sequencing on individual basis according to standard procedures for 17 SNVs with various characteristics (having different AF, being detected in only 1 to 5 pools and being known/unknown in public databases). For only 25% of the SNVs detected in the pools we were able to confirm them in the individual patient. It appeared that these false-positives SNVs were unbalanced for Forward/Reverse (F/R) sequencing reads. This was particularly true for the very rare SNVs detected in only one pool most likely as a result of PCR artefacts created during the Haloplex target enrichment. However, by selecting only SNVs with F/R balance between 20-80%, we were certain to exclude these PCR artefacts. For variants present in only one pool, we used a stricter F/R balance between 40-60%.

*Imputation* Immunochip genotyping available for approximately 50% of the 790 UC patients that were sequenced in the discovery part of the study. We performed imputation of these 437 samples using the population matched Genome of The Netherlands as a reference panel[3,4]. Before imputation, the Immunochip genotypes where filtered on SNPs with a MAF above 0.001, a Hardy Weinberg Equilibrium p > 10^-4^ and call rate of 0.9. After quality control the data was pre-phased using SHAPEIT2[5]. The phased genotypes were aligned to the GoNL reference panel, using Genotype Harmonizer[6]. The imputation was performed using Impute2 version 2.3.0[7]. We used the MOLGENIS compute imputation pipeline to generate our scripts and monitor the imputation. **(S2 Table)**

**Phase II: Replication phase 1**

**Quality control**

After genotyping of 171 SNVs in 1053 Dutch UC cases and 1170 Dutch UC controls with 5 Agena Bioscience iPlexes, quality control was perfomed with PLINK v1.07 software. SNVs with a Hardy–Weinberg equilibrium p < 0.0001 in controls and an overall call rate < 90% were excluded. Individuals with < 50% of SNVs confidently genotyped were excluded. The dataset after quality control consisted of 1021 UC cases, 1166 healthy controls and 111 SNVs, with a genotype call rate of 98% **(S2 Table).**

**Phase III: Replication phase 2**

**Quality control**

Genotyping of 19 SNVs was performed in 1064 German UC cases and 3576 general population-based German controls with the iPlex Agena Bioscience system (<http://agenabio.com>) .For quality control, SNVs with a Hardy–Weinberg equilibrium p < 0.0001 in controls and an overall call rate < 90% were excluded. Individuals with < 50% of SNVs confidently genotyped were excluded. The dataset after QC consisted of 1027 UC cases, 3532 healthy controls and 17 SNVs, with a genotype call rate of 99%.

**Supplementary Blasting and Cluster plot example**

**SNP rs41376152**

Chr: position 11:1094761

Gene: MUC2

Blating results: (<http://genome.ucsc.edu/cgi-bin/hgBlat0>)

Blat results (1)

Identity 100.0%

Chr 11

Start 1094715

End 1094795

Blast sequence:

AGCACTGTGC AGACGACCAC* CACCAGTGCC TGGACCCCAA CGCCGA[C]^#^CCC ACTCTCCACA CCCAGCATCA TCAGGACCAC A*

*=Sequence with underscore is designed primers

#= Single nucleotide variant (SNV)


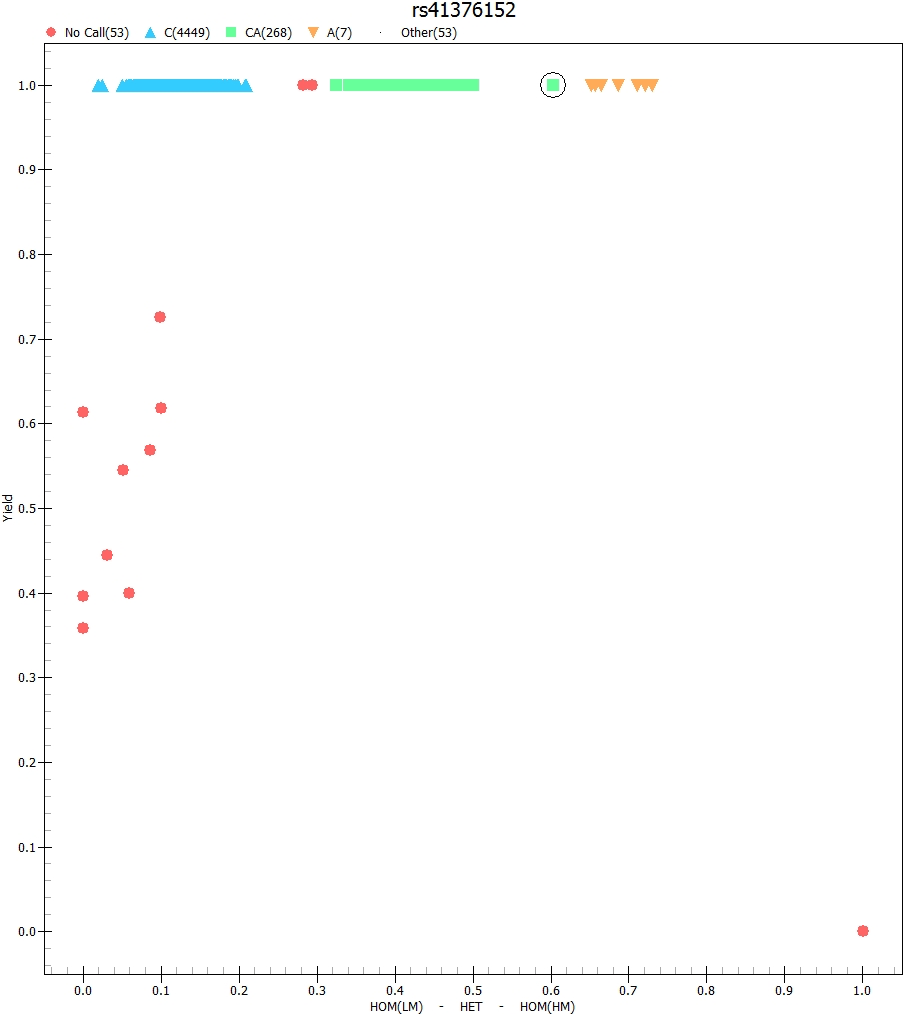


Clusterplot rs41376152 generated with AgenaBioscience Typer Software.

**Supplementary References**

1. Jostins L, Ripke S, Weersma RK, Duerr RH, McGovern DP, Hui KY, et al. Host-microbe interactions have shaped the genetic architecture of inflammatory bowel disease. Nature. Nature Publishing Group; 2012;491: 119–124. doi:10.1038/nature11582

2. Mizoguchi A, Mizoguchi E. Animal models of IBD: Linkage to human disease. Curr Opin Pharmacol. Elsevier Ltd; 2010;10: 578–587. doi:10.1016/j.coph.2010.05.007

3. Francioli LC, Menelaou A, Pulit SL, van Dijk F, Palamara PF, Elbers CC, et al. Whole-genome sequence variation, population structure and demographic history of the Dutch population. Nat Genet. Nature Publishing Group, a division of Macmillan Publishers Limited. All Rights Reserved.; 2014;advance on. doi:10.1038/ng.3021

4. Deelen P, Menelaou A, van Leeuwen EM, Kanterakis A, van Dijk F, Medina-Gomez C, et al. Improved imputation quality of low-frequency and rare variants in European samples using the “Genome of The Netherlands”. Eur J Hum Genet. Macmillan Publishers Limited; 2014;22: 1321–6. doi:10.1038/ejhg.2014.19

5. Delaneau O, Zagury J-F, Marchini J. Improved whole-chromosome phasing for disease and population genetic studies. Nat Genet. Nature Publishing Group, a division of Macmillan Publishers Limited. All Rights Reserved.; 2013;10: 5–6. doi:10.1038/nmeth.2307

6. Deelen P, Bonder MJ, van der Velde KJ, Westra H-J, Winder E, Hendriksen D, et al. Genotype harmonizer: automatic strand alignment and format conversion for genotype data integration. BMC Res Notes. 2014;7: 901. doi:10.1186/1756-0500-7-901

7. Howie BN, Donnelly P, Marchini J. A flexible and accurate genotype imputation method for the next generation of genome-wide association studies. PLoS Genet. Public Library of Science; 2009;5: e1000529. doi:10.1371/journal.pgen.1000529
